# Intracardiac thrombus in Behçet's disease: Two case reports

**DOI:** 10.1186/1477-9560-3-9

**Published:** 2005-07-25

**Authors:** Sonia Hammami, Silvia Mahjoub, Khaldoun Ben-Hamda, Radhia Brahem, Habib Gamra, Mohamed Ben Farhat

**Affiliations:** 1 Department of Internal Medicine, F Bourguiba University Hospital, Monastir, Tunisia; 2 Department of Cardiology, F Bourguiba University Hospital, Monastir, Tunisia; 3 Department of Radiology, F Bourguiba University Hospital, Monastir, Tunisia

**Keywords:** Intracardiac thrombus, Behcet's disease

## Abstract

Intracardiac thrombus in Behçet's disease is an extremely rare manifestation. We report two such cases. A 20-year-old man presented with dyspnoea, cough and haemoptysis. Right heart thrombus associated with pulmonary artery aneurysm and thromboembolism was identified by helical CT and transoesophageal echocardiography. The second case was a 29-year-old male admitted for fever and chest pain. A diagnosis of right atrial thrombosis associated with pulmonary embolism and hyperhomocysteinemia was made. Due to the absence of haemodynamic compromise, medical management consisting of immunosupressive and anticoagulation therapy was adopted which resulted in complete dissolution of the thrombus with dramatic clinical improvement in both cases of clinical status.

**Conclusion:** intracardiac thrombus is a rare complication of Behçet's disease. As shown in our patients, medical treatment should be considered as the first line.

## Background

Behcet's disease (BD) is a multisystemic inflammatory disease with a clinical spectrum that has greatly expanded since it was first described in 1937 as a triple complex of recurrent oral genital ulcers and uveitis. Cardiac involvement is extremely rare and often associated with poor prognosis [1]. We report two patients with a large intracardiac thrombus (ICTs) out of a series of 130 patients with BD.

## Case reports

### Case 1

A 20-year-old Tunisian man with a three weeks history of dyspnoea, cough and haemoptysis was admitted. At the age of 18, he had suffered from painful oral and genital ulcerations and polyarthralgias. At that time, examination revealed bilateral papillary oedema and brain magnetic resonance imaging showed superior sagittal and left lateral thromboses. The patient was given oral prednisone at the dose of 1 mg/kg/day that was tapered gradually and colchicine 1 mg/day in addition to acenocoumarol to maintain International Normalized Ratio between 2 and 3. These medications were discontinued per the patient 7 months later which resulted in the recurrence of aphtous ulcerations and papulopustular eruptions episodes.

On physical examination the patient had fever, face and neck oedema, prominent superficial thoracic venous collaterals, and pseudofolliculitis lesions. There was evidence of penile and scrotal scarring and minor aphthae on the buccal mucosa. Blood pressure was 110/80 mm Hg and pulse rate 100/min.

**Figure 1:**
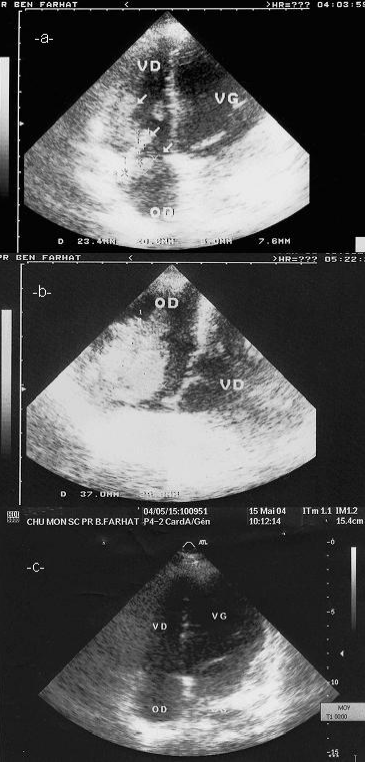
Transthoracic echocardiography: in apical four chamber view. (VD: right ventricle, VG: left ventricle, OD: right atrium, OG: left atrium) image of one thrombus in the right atrium, and two thrombi in the right ventricle **(a)**, transoesophageal echocardiography showing a large thrombus **(b)**, after treatment, complete resolution of the thrombus **(c)**.

**Figure 2:**
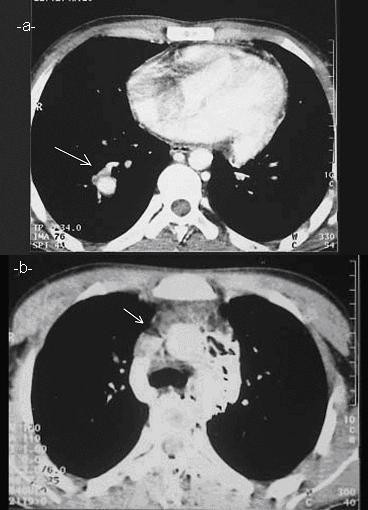
Chest helical computed tomography demonstrating a single (14 mm) right main pulmonary artery aneurysm and image of thrombi in the right heart (**a**), diffuse venous collateral vessels and superior vena cava thrombosis (**b**).

As to laboratory tests, haemoglobin concentration was of 9 g/dl, erythrocyte sedimentation rate of 60 mm/hr and C reactive protein concentration of 19 mg/l (normal < 2 mg/l). Renal and liver function tests were normal. Levels of serum IgG and IgM anticardiolipin, protein C, protein S, antithrombine III and total homocysteine were within the normal range. Electrocardiogram showed sinus rhythm tachycardia with no other abnormalities. Chest X-ray demonstrated hilar enlargement. Transthoracic and transoesophageal echocardiography showed multiple cardiac masses, one in the right atrium protruding through the tricuspid valve and two in the right ventricle (Figure 1 – see Additional file 1). Helical computed tomography (CT) showed multiple thrombi in both right atrium and ventricle extending into the superior vena cava. There was innominate and brachiocephalic vein occlusion, it also showed bilateral pulmonary embolism and multiple pulmonary infarcts in the lower lobe of the lungs. A large (14 mm in diameter) aneurysm located in the right basal segmental arteries (Figure 2 – see Additional file 2). We opted for a treatment based on low molecular weight heparin twice daily then oral anticoagulant, 1 gr of methyl prednisolone per day for 3 days, 1 mg/kg/day of oral, then tapered over 3 weeks and 1 g pulse cyslophosphamide monthly associated with colchicine 1 mg/day.

Two weeks later, oedema of the chest and neck has completely resolved. Thrombus size has substantially decreased. Nine months after discharge, no cardiac masses were detected by echocardiography and CT scan showed no evidence of previously mentioned thromboses with a complete disappearance of pulmonary aneurysm.

### Case 2

**Figure 3:**
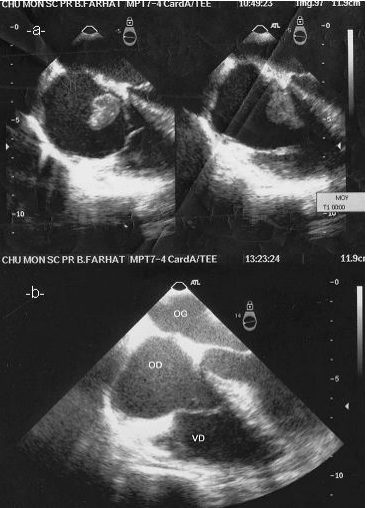
Transthoracic echocardiography: image of the thrombus in the right atrium (**a**), after treatment complete resolution of the thrombus (**b**).

A 29-year-old Tunisian man with a two-month history of fever of unknown origin, weight loss and inspiratory thoracic pain was admitted. He had suffered from both genital and oral ulcers over five months. The initial physical examination revealed a temperature of 38°C, multiple pseudofolliculitis, oral and scrotal ulcerations. Laboratory tests on admission revealed: haemoglobin of 13 g/dl, erythrocyte sedimentation of 105 mm/hr and C reactive protein concentration of 204 mg/l. The tests looking for antiphospholipid antibodies, protein C, protein S and antithrombine III deficiencies were negative. The plasma total homocysteine level was 27 μmol/l (normal < 10 μmol/l). HLAB5 and pathergy tests were positive. Electrocardiogram showed sinus rhythm tachycardia, and chest X-ray was normal. Transoesophageal echocardiography revealed a cardiac mass in the right atrium of 20/23 mm size attached into atrial septum protruding through the tricuspid valve into the right ventricle (Figure 3 – see Additional file 3). These findings were confirmed by CT scan that also showed a partial obstruction of the terminal portion of the inferior vena cava and thrombosis of the left lobar pulmonary artery with multiple pulmonary infarcts. We started treatment with intravenous heparin then oral anticoagulant, 1 gr of methyl prednisolone per day for 3 days, 1 mg/kg/day of oral, which was tapered gradually and 1 g pulse of cycslophosphamide monthly associated with colchicine 1 mg/day. Consequently, the thrombus in the right atrium has substantially decreased in size. At five months follow-up, a complete resolution of the thrombi in the right atrium, vena cava and pulmonary artery tree was observed.

## Discussion

Cardiac manifestations in BD which are indication of poor prognosis were reported to occur in about 1 – 5% of cases [1]. They consist of cardiomegaly, endocarditis or pericarditis and less commonly of myocardial infarction and myocarditis [2,3]. Association with intracardiac thrombus which is a serious complication, is even more rare, up to this date, less than 50 cases have been reported so far [4-8].

Our patients fulfilled the proposed criteria of the international study group for BD [9], with active disease (two or more active clinical features related to BD). Two interesting issues about these cases should be emphasized: first, the unusual presentation of BD with ICTs and pulmonary thromboembolism, second, the favourable response to medical management. The association of intracardiac thrombosis, with the less uncommon pulmonary arteritis and vena cava thrombosis was described for the first time by Houman [10] and reported in only few cases so far [4]. In our patients, we can reasonably exclude retrospectively myxoma and endocarditis, in view of the mass resolution on immunosuppressive and anticoagulation therapy.

Biopsy carries an excessive risk [2], but has the advantage of providing material for histological examination. The organized thrombus usually contained an inflammatory cell infiltrate composed of a mixture of granulocytes and mononuclear inflammatory cells or predominantly lymphocytes. The histologic descriptions of the thrombi may be dependent on the biopsy timing [4]. We did not perform a right ventricular biopsy in our patients.

The pathogenic mechanism underlying thrombotic tendency in patients with BD is not well known. It is however believed to be due to endothelial cell ischemia or disruption that leads to enhancement of platelet aggregation [11]. Also decreased release of vascular tissue plasminogen activator has been reported in systemic and cutaneous vasculitis [12]. Another possible pathogenic mechanism of thrombosis in BD is attributed to the presence of anti phospholipid antibodies which is reported to be present in 18% of cases [13,14]. Elevated Von Willebrand factor antigen levels have recently been demonstrated [15]. Hyperhomocysteinemia was reported to be present in patients with BD and was associated with increased risk of vascular thrombosis [16], which may have contributed to cardiac thrombus formation in our second patient. To the best of our knowledge this association has not been previously reported.

Diagnosis may be confirmed either at necropsy or after surgery. In our cases, this complication of BD was diagnosed by echocardiography. Other imaging modalities including CT scan and MRI can show vascular complications and give information concerning the lung parenchyma. The management of ICTs is still controversial [4]. Surgical removal has the advantage of providing material for histological examination; medical management however was associated with a better outcome. Mogulkoc's review about 24 cases of ICTs, surgery was unsuccessful in 4/12 cases, whereas complete resolution of the thrombus on medical therapy was observed in 7/8 [4].

Anticoagulant or thrombolytic therapy was the first line treatment of intracardiac thrombus [4]. In the presence of pulmonary aneurysms this therapy could lead to fatal haemoptysis especially in bilateral and large aneurysms. In our first case ICTs was associated with multiple venous thrombi and only a small aneurysm with no haemodynamic compromise. Considering the risk of surgical treatment we preferred a conservative approach with immunosuppressive and anticoagulant therapy.

We conclude that thrombi especially in the right heart cavities can be present in BD without causing specific symptoms but can lead to pulmonary embolism. Early echocardiography seems advisable to detect the presence of cardiac involvement and medical therapy can be effective in the resolution of ICTs in the setting of BD.

## List of abbreviations used

BD: Behcet's disease

ICTs: IntraCardiac Thrombus

CT: Helical Computed Tomography

## Competing interests

The author(s) declare that they have no competing interests.

## Authors' contributions

S.H.: conceived of the study, tacked out the responsibility for diagnosis of patients, their evolution and elaborated in the design of these 2 cases and draft the manuscript; S.M. carried out the therapy evolution; K.B.H.: interested in the cardiac aspects and echocardiography diagnosis; R.B.: tacked out X-ray, MRI and CT examinations; H.G and M.B.H: revised the article critically for important intellectual content and have given final approval of the version to be published. All authors read and approved the final manuscript.
